# IL-33 Contributes to *Schistosoma japonicum*-induced Hepatic Pathology through Induction of M2 Macrophages

**DOI:** 10.1038/srep29844

**Published:** 2016-07-21

**Authors:** Hui Peng, Qixian Zhang, Xiaojuan Li, Zhen Liu, Jia Shen, Rui Sun, Jie Wei, Jia Zhao, Xiaoying Wu, Feng Feng, Shuping Zhong, Xi Sun, Zhongdao Wu

**Affiliations:** 1 Key Laboratory of Tropical Disease Control (Sun Yat-sen University), the Ministry of Education PRC, Guangzhou, 510080, China; 2 Zhongshan School of Medicine, Sun Yat-sen University, 74 Zhongshan 2nd Road, Guangzhou, 510080, China; 3 School of Pharmaceutical Sciences, Southern Medical University, 1838 North Guangzhou Avenue, Guangzhou, 510515, China; 4 The Third Affiliated Hospital of Sun Yet-sen University, Guangzhou, 510630, China; 5 Department of Biochemistry and Molecular Biology, Keck School of Medicine, University of Southern California, 2011 Zonal Avenue, HMR 605, Los Angeles, CA 90033, USA

## Abstract

Interleukin (IL)-33 is involved in T helper (Th)2-biased immune responses in mice infected with *Schistosom*a, but the precise mechanism remains to be elucidated. Herein, we investigated the role of IL-33 and its receptor ST2L in hepatic granuloma pathology induced by *Schistosoma japonicum* infection. We found that IL-33 induced the increased production of IL-5 and IL-13 from splenocytes and liver mononuclear cells (MNCs) of infected mice. The infected mice developed significantly higher number of ST2L-expressing cells in spleen and liver. Most of the ST2L-expressing cells in liver were F4/80^+^ macrophages, indicating the key role of macrophages in the response to IL-33. However, the liver MNCs in male-only worm infection had a poor response to IL-33, though elevated serum IL-33 was observed. ST2L^+^F4/80^+^ cells were lower in male-only worm infection than that of mixed infection. IL-33 and soluble egg antigen (SEA) upregulated ST2L expression on macrophages *in vitro* and ST2L-expressing macrophage displayed MHCII^-^CD11b^+^M2 phenotype. Macrophage deletion significantly attenuated IL-33-induced type 2 immunity and egg granuloma formation during *S. japonicum* infection. These data demonstrate that IL-33 contributes to hepatic granuloma pathology through induction of M2 macrophages during *S. japonicum* infection.

Fibrosis of the liver is a consequence of chronic liver disease caused by a several factors including infection. The parasitic disease schistosomiasis and resulting hepatitis are often associated with hepatic fibrosis accompanied by significant morbidity and mortality in Asian countries^1^,^2^. *Schistosoma japonica* is an important cause of hepatic fibrosis in endemic areas of Asia. The fibrosis is thought to be the result of the deposition of large number eggs in the liver of humans by the parasite^3^. The deposited eggs in host liver triggers the formation of granulomata that lead to chronic fibrosis. Thus, better understanding of the mechanistic basis of granuloma formation is important to prevent this infection-associated pathology.

Experimental models of hepatic schistosomiasis clearly demonstrate that host immune responses are essential for the development of granulomatous pathology^4^,^5^,^6^. In *S. japonicum*-infected mice, a T helper (Th)1 response is initiated early in infection followed by an interleukin (IL) 4 and IL-13-mediated dominant Th2 immune response as eggs become lodged in the host liver. At this time macrophages in the egg-induced granuloma are polarized into a M2 macrophage phenotype^7^,^8^. These macrophages, known as alternatively activated macrophages (AAM), contribute to the development of fibrosis, maintenance of granuloma, tissue repair, and host survival^9^,^10^. Not surprisingly, in animal models of this infection by male or female *Schistosoma* cercariae alone, the potent Th2-inducing properties are absent since there is no schistosome egg production in single infection.

IL-33, a ligand for the orphan IL-1 family receptor ST2L, has been implicated in initiating, amplifying, and maintaining Th2 responses and M2 macrophages^11^,^12^,^13^. IL-33 and ST2L are constitutively expressed in healthy liver and their expression levels are increased in hepatic ischemia/reperfusion^14^. Recently, Arshad *et al*. reported that IL-33 was constitutively expressed in liver vascular endothelial cells and sinusoidal endothelial cells^15^. It has been demonstrated that the hepatocyte is a major source of IL-33 in acute murine hepatitis^16^,^17^. However, little is known regarding the role of IL-33 in the formation of granuloma in the liver during *S. japonicum* infection.

We recently reported that mice infected with the nematode parasite, *Angiostrongylus cantonensis*, displayed increased expression of IL-33^18^. Given the importance of IL-33 in type 2 responses, the current study was undertaken to investigate the role of IL-33 in *S. japonicum* infection. Herein, we examined the role of IL-33 in regulating host immune responses and liver pathology during *S. japonicum* infection. We observed that infected mice developed significantly higher number of ST2L-expressing cells in spleen and liver. Most of the ST2L-expressing cells in liver were F4/80^+^ macrophages, indicating that macrophages are the dominant cell-type responsive to IL-33. However, the liver MNCs in mice with male-only worm infection displayed a poor response to IL-33, though elevated serum IL-33 was observed. The numbers of ST2L^+^F4/80^+^ cells were lower in male-only worm infections than in mixed infection. Additionally, IL-33 and soluble egg antigen (SEA) up-regulated ST2L on macrophages and ST2L^+^ macrophage I^-^CD11b^+^M2 phenotype. Our studies further demonstrate that hepadisplayed an MHCItic granuloma formation was significantly attenuated in mice following macrophage depletion during *S*. *japonicum* infection.

## Results

### *S. japonicum* infection drives IL-33 expression

**Figure 1:**
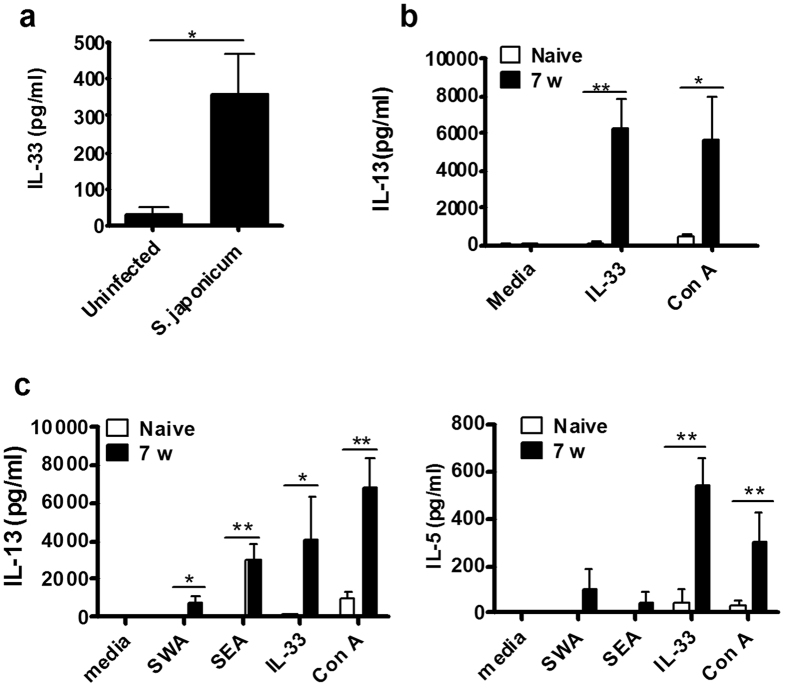
*S. japonicum* infection promoted IL-33 expression and type 2 cytokine production. (**a**) Serum IL-33 in healthy controls (n = 20) or patients with *S. japonicum* infection (n = 23) was determined by ELISA (*p < 0.05). (**b**) Liver mononuclear cells (MNCs) from naive mice or from *S. japonicum*-infected mice at week 7 post-infection were activated with IL-33 (10 ng/ml) or ConA (5 μg/ml) for 72 hours. Supernatants were collected for IL-13 ELISA assay. (**c**) Splenocytes from naive mice or from *S*. *japonicum*-infected mice at week 7 post-infection were activated with SWA (50 μg/ml), SEA (20 μg/ml), IL-33 (10 ng/ml) or ConA (5 μg/ml) for 72 hours. Supernatants were collected for IL-13 and IL-5 ELISA. Data represent observations from 3 independent experiments and are shown as mean ± standard error of the mean (SEM), *p < 0.05 and **p < 0.01, by the Student t test.

Previously, our studies strongly suggested that IL-33 was important for type 2 immune responses induced by parasites^18^. To investigate whether IL-33 is also involved in the pathogenesis of schistosomiasis, we measured serum levels of IL-33 obtained from patients infected with *S. japonicum.* As shown in Fig. 1a, significantly higher levels of IL-33 were observed in patients infected with *S*. *japonicum* compared to uninfected controls (*p* < 0.05).

Since IL-33 regulates type-2 cytokines^19^, we examined its role in modulating cytokine production in BALB/c mice infected with *S*. *japonicum* and euthanized at week 7 post-infection. Splenocytes were cultured in the presence or absence of schistosome worm antigen (SWA), schistosome egg antigen (SEA), IL-33 or Concanavalina (Con A) for 72 hours and supernatants assayed by ELISA. We found that IL-33 significantly increased levels of IL-5 and IL-13 in splenocytes obtained from infected mice (Fig. 1c), whereas splenocytes isolated from uninfected mice did not respond to IL-33 *in vitro*; neither IL-5 nor IL-13 were detected. Interestingly, IL-33 induced much higher levels of IL-5 and IL-13 from splenocytes compared to SWA and SEA. We also observed that there was a significant increase in IL-13 production by IL-33 stimulated liver cells in infected mice compared with liver cells obtained from uninfected mice (Fig. 1b).

### IL-33 promotes type 2 cytokine production and liver pathology only in mixed male and female infection

**Figure 2:**
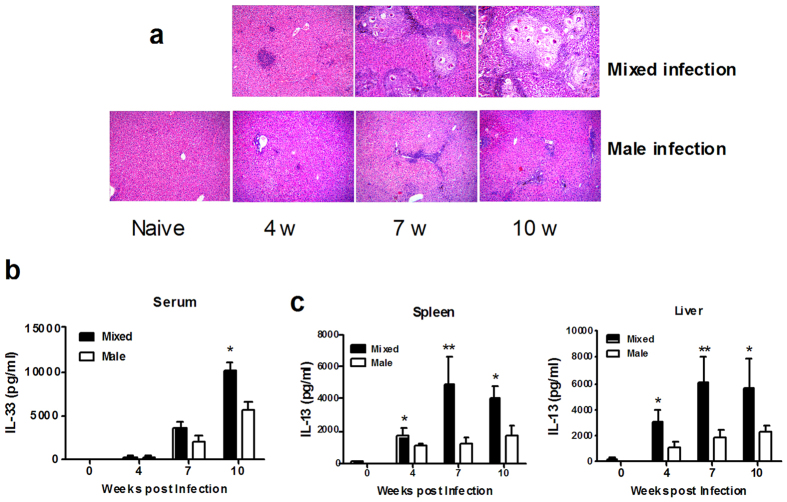
IL-33 enhanced IL-13 production and liver pathology in S. *japonicum*-infected mice only in egg-phages. (**a**) H&E staining of liver sections (Magnification 10X). (**b**) Serum IL-33 was analyzed by ELISA at weeks 4, 7 and 10 post-infection. (**c**) Liver MNCs were purified from the liver of BALB/c mice with a mixed sex or a male-only worm infection at weeks 4, 7, 10 post-infection and activated with IL-33 (10 ng/ml) for 3 days. The supernatants were collected and IL-13 production was detected by ELISA. Data indicate mean ± SEM of 3–5 mice per group; *p < 0.05 and **p < 0.01, by the Student t test.

We next determined the role of IL-33 in the formation of liver granuloma. BALB/c mice were infected with conventional mixed male and female *S. japonicum* where eggs were laid (mixed sex infection) or with a male-only infection which there were no eggs (male-only infection). Granuloma formation in the liver with mixed sex infection is shown in Fig. 2a. We then compared the expression level of IL-33 in serum from mixed sex infection and male-only infection mice at 4, 7, and 10 weeks post-infection. Although, a significant increase in IL-33 was detected at weeks 7 and 10 post infection (*P* < 0.01, as compared to week 4 post infection), there were no significant difference in the serum levels of IL-33 between mixed sex infection and male-only infection mice at week 7 post infection (Fig. 2b). However, IL-33 induced higher levels of IL-13 production in spleen and liver of mice with mixed infected than in male-only infection (Fig. 2b). Taken together, these observations suggest that SEA regulates the expression of the receptor of IL-33, although the expression of IL-33 itself is independent on SEA.

### Macrophages are the major cell-type-responsive to IL-33 in the spleen and liver granuloma of *S. japonicum*-infected mice

**Figure 3:**
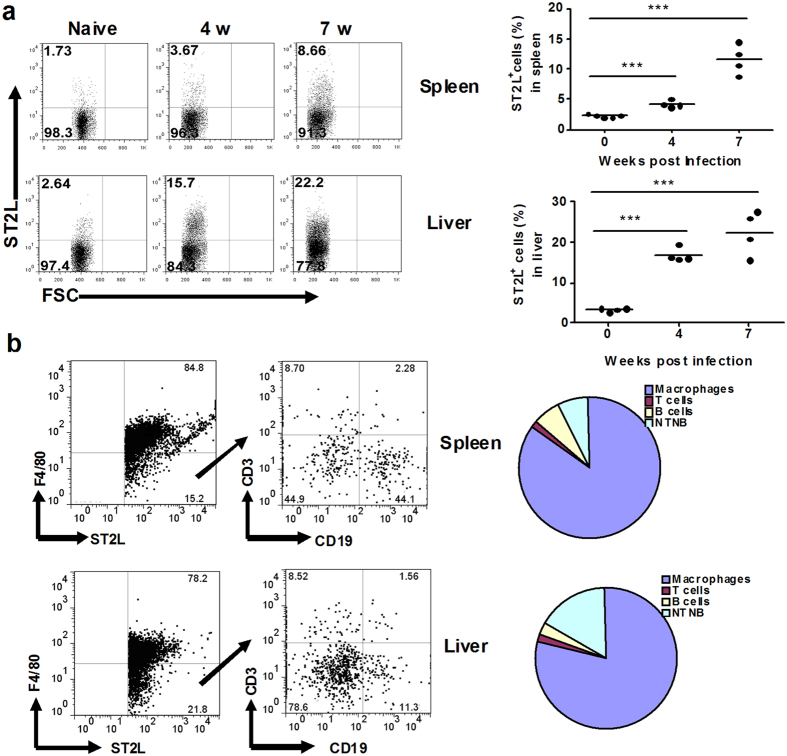
Macrophages are the major cell-type-responsive to IL-33 in spleen and liver granuloma of *S. japonicum* infected mice. (**a**) Increased expression of ST2L on splenocytes and liver MNCs from S. *japonicum*-infected mice. Splenocytes and liver MNCs were isolated from the infected mice at week 0, 4 and 7 post-infection and detected ST2L by flow cytometry. High levels of ST2L were expressed on cells of mice infected with *S*. *japonicum.* The percentages of positive cells are indicated at the upper left of each chart. (**b**) ST2L^+^ cells could be expressed on the surface of different cell populations, including, F4/80^+^ macrophages, CD3^+^ T cells, CD19^+^B cells and CD19^-^CD3^-^NTNB cells. High levels of ST2L were expressed on the surface of macrophages. The flow cytometry data are representative of two independent experiments; *p < 0.05 and **p < 0.01, by the Student t test.

IL-33 targets cells of immune system via ST2L^13^. In order to detect ST2L expression on cells from spleens and livers during *S*. *japonicum* infection at different time points, BALB/c mice were infected and euthanized at weeks 4, 7, and 10 post-infection, Splenocytes and liver MNCs were prepared for FACS analysis. We demonstrated that ST2L^+^ cells were upregulated on both splenocytes and liver MNCs after infection (Fig. 3a). Since previous data demonstrated that T cells producing type 2 cytokines express ST2L on their surface both *in vitro* and *ex vivo*^20^, we first detected ST2L expression on CD4^+^ T cells during infection. However, we found that the frequency of ST2L-expressing CD4^+^ cells was increased in liver granuloma (10 ± 2% of CD4^+^ cells *vs* 0.6 ± 0.1% in control livers) with no significant upregulation in the spleen (data not shown). These observations indicate that the receptor of IL-33 was upregulated on cells other than CD4^+^ cells, which led us to further identify which cell population was the dominant IL-33-responsive cell in schistosome egg granuloma.

In order to define the cells in schistosome egg granulomas responsible for expressing ST2L, the infected BALB/c mice were euthanized at week 7 post infection and single-cell suspension of liver and spleen were stained with surface markers against macrophages, T cells and B cells as well as ST2L. We demonstrated that specific ST2L could be expressed in different cells. More than 75% of ST2L^+^ cells were F4/80^+^ macrophages in liver granuloma, while 25% of ST2L-expressing cells were either T or B cells as well as non-T or B cells (Fig. 3b). More than 75% of ST2L-expressing cells were F4/80^+^ macrophages in spleen. These observations indicate that macrophages are the major source of ST2L in the spleen and liver granulomas and the type 2 cytokines induced by IL-33 are dependent on the macrophage during *S. japonicum* infection.

### Expression of ST2L on macrophages in the liver from male-only infected mice was impaired

**Figure 4:**
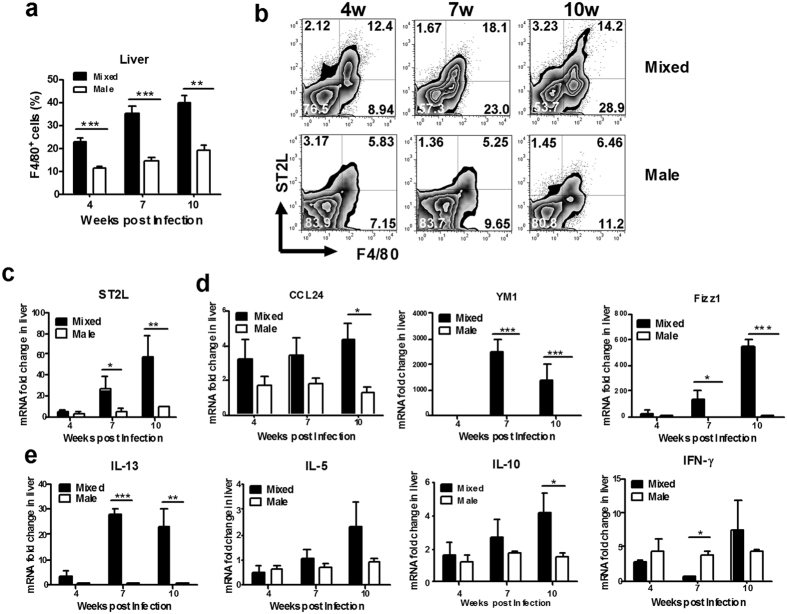
Expression of ST2L on macrophages in the liver from male-only infected mice was impaired. (**a**) Liver MNCs from infected mice were stained with anti-F4/80^-^FITC. The percentage of F4/80^+^ cells was higher in mice with mixed infection than with male-only infection. (n = 3–4 for each group; **p < 0.01, ***p < 0.001). (**b**) Note the significant increase in the total number of ST2L^+^F4/80^+^ cells in livers of mixed infected mice compared with male-only infected mice at weeks 4, 7, 10 post-infection. (**c**) mRNAs of ST2L in liver total RNA were quantified by qPCR (n = 3 per group, *p < 0.05, **p < 0.01). (**d**) mRNAs of selected Th1/Th2 cytokines in liver total RNA were quantified by qPCR (n = 3 per group, *p < 0.05, **p < 0.01, ***p < 0.001). (**e**) mRNAs of selected M2 cytokines in the liver total RNA were quantified by qPCR (n = 3 per group, *p < 0.05, **p < 0.01, **p < 0.001). Data are representative of two separate experiments.

Schistosoma infection results in a prominent Th2 response when egg production is initiated^4^. We found that more macrophages were recruited to the livers in mixed infection than in male-only infection (Fig. 4a). Next, we compared ST2L-expressing macrophages in the livers of mixed infection with male-only infection. As shown in Fig. 4b, there were more ST2L^+^ macrophages in the liver of mixed infection than male-only infection at weeks 4, 7, and 10 post infection. Using quantitative RT-PCR, we detected significantly increased expression of ST2L in the livers of mixed infected mice compared to male-only infected mice (Fig. 4c). Furthermore, we found that mRNA levels of Th2 cytokines (IL-13 and IL-10) were significantly higher in mixed infected mice than male-only infected mice (Fig. 4e). Conversely, the expression of IFN-γ, a Th1 cytokine, was lower in mixed infected mice than male-only infected mice at week 7 post-infection (Fig. 4e). Taken together, these data indicate that SEA enhances ST2L expression and promotes type 2 immune responses.

IL-33 enhances the polarization of macrophage toward M2 phenotype^12^,^21^. Consistent with these findings, the expression of Ym-1 and Fizz1, two molecules associated with M2, were much higher in livers of mixed infected mice than male-only infected mice. Higher CCL24 expression was also observed in livers of mixed infected mice compared to male-only infected mice (Fig. 4d).

### Both SEA and IL-33 promote ST2L expression and enhance the polarization of AAM *in vitro*

**Figure 5:**
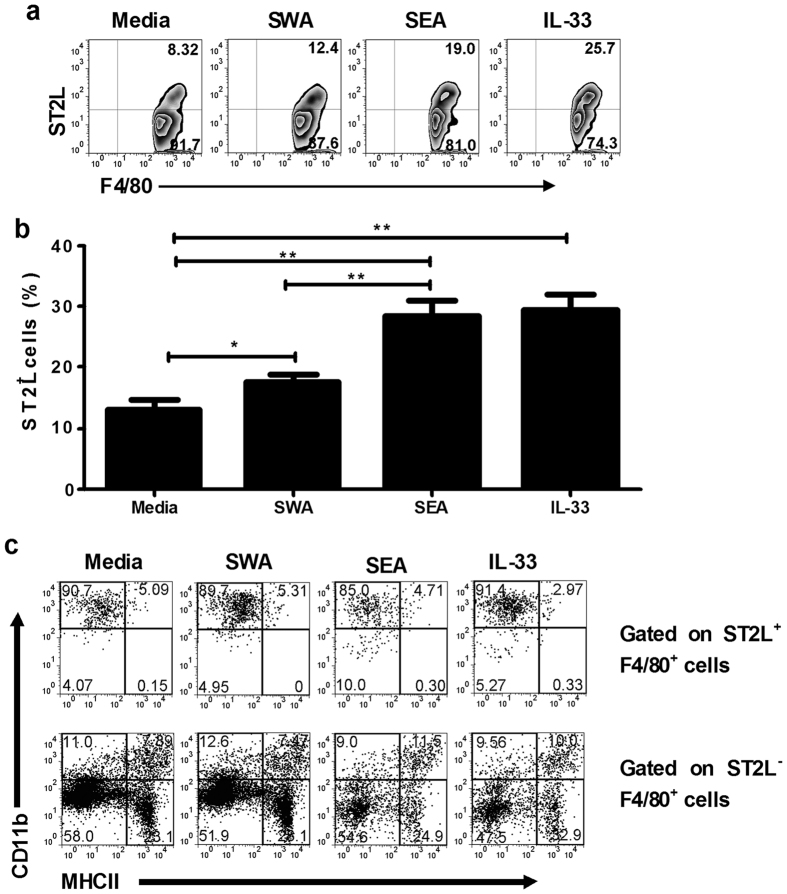
Both SEA and IL-33 promote ST2L expression and enhance the polarization of AAM. Liver MNCs from mice with male-only infection for 4 weeks were stimulated with SWA (50 μg/ml), SEA (20 μg/ml), IL-33 (10 ng/ml) for 48 h before the surface markers staining. Cells were stained for ST2L, F4/80, MHCII, and CD11b and analyzed by FACS gated on F4/80^+^ cells. (**a**) The number is the percentage of positive cells, respectively. Data are representative of three separate experiments. (**b**) The frequency of ST2L^+^F4/80^+^ cell from three different experiments is summarized. Data indicate mean ± SD (*p < 0.05, **p < 0.01). (**c**) Expression of CD11b and MHCII were analyzed when cells in (**a**) were gated on ST2L^+^F4/80^+^ cells or ST2L^−^F4/80^+^ cells. Quadrants and numbers in indicate percentage of cells in gated ST2L^+^F4/80^+^ cells or ST2L^−^F4/80^+^ cells. Data are representative of three separate experiments.

Given the well-established contribution of IL-33/ST2L to type 2 immune responses^12^,^18^ and the expression of ST2L on macrophages^21^, we reasoned that IL-33 might be involved in AAM macrophage activation. To characterize the effect of IL-33 on macrophage activation, we prepared liver MNCs and cultured MNCs with IL-33(10 ng/ml), SEA (20 μg/ml), and SWA (50 μg/ml) for three days before analyzing the number of ST2L-expressing macrophages. We demonstrated that both IL-33 and SEA caused an increase in the levels of ST2L expression on macrophage compared to SWA (Fig. 5a,b). We further examined the phenotype of ST2L^+^ and ST2L^−^ macrophage and found that ST2L^+^ macrophage displayed significantly increased levels of CD11b and low levels of MHCII (Fig. 5c), displaying an M2 phenotype^22^.

### Macrophage depletion *in vivo* impairs IL-33-induced type 2 immunity

**Figure 6:**
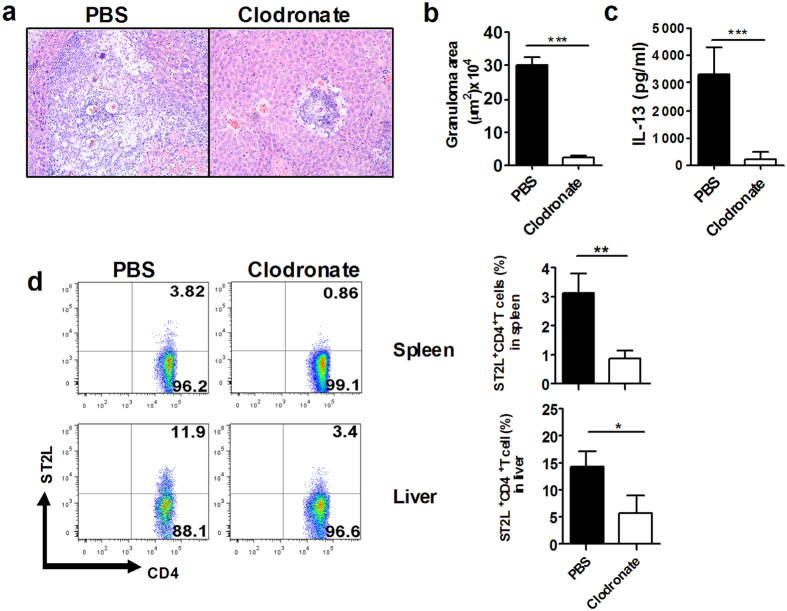
Macrophage depletion in mice infected with *S. japonicum* impaired IL-33-induced type 2 immunity and hepatic egg granuloma formation. (**a**) Histopathology of *S. japonicum*-infected mice treated with PBS or clodronate. Liver sections stained with H&E show a granuloma surrounding parasite eggs (Original magnification, X10). (**b**) Average granuloma sizes in infected mice treated with PBS or clodronate (***p < 0.001). (**c**) IL-33-induced upregulation of IL-13 was attenuated in spleen in mice treated with PBS or clodronate. (n = 5 per group, ***p < 0.001). (**d**) CD4^+^ST2L^+^ cells in liver and spleen from infected mice treated with PBS or clodronate (n = 3–4 mice per group; *p < 0.05, **p < 0.01).

After establishing that macrophages were capable of responding to IL-33 and producing type 2 cytokines, we investigated if macrophages were essential for the *in vivo* induction of type 2 immunity in response to IL-33. Two groups of mice were treated with clodronate-containing liposomes to deplete macrophages or PBS as controls at 4-6 weeks after infection. Clodronate treatment decreased the number of tissue macrophages, as shown by the significantly decreased expression of F4/80^+^ cells in spleen. Correspondingly, the IL-33-induced IL-13 cytokine response in spleen (Fig. 6c) was significantly attenuated in mice treated with clodronate compared to PBS-treated controls, although, there was no significant difference in serum IL-33 levels between these two groups (data is not shown). In addition, there was a significant reduction in liver egg granuloma in mice treated with clodronate compared to PBS-treated controls (Fig. 6a,b). Interestingly, we found that ST2L^+^CD4^+^ cells were decreased in both spleen and liver of mice treated with clodronate compared to PBS-treated controls (Fig. 6d). Overall, these data indicate that macrophages are essential for the development of type 2 immunity induced by IL-33.

## Discussion

IL-33 contributes to the initiation of type-2 immune responses following infection with several pathogens and allergens. In liver, IL-33 plays an important role in viral and immune cell mediated pathology^15^,^23^. In schistosomiasis, the immune response against schistosome eggs results in granuloma formation in liver and initiates the Th2-biased immunity that prevents acute mortality of host but ultimately causes liver fibrosis^4^. Here, we investigated the role of IL-33 during *S. japonicum* infection. We demonstrated increased levels of IL-33 in the serum in both infected human and mice, consistent with a recent study reporting an association of IL-33 overexpression with *S. mansoni* dependent liver damage^17^. Therefore, we examined the possibility that this cytokine also plays a role in regulating type 2 responses and/or alternative activation of macrophage and coincident development of egg granuloma pathology during *S. japonicum* infection.

We found that both liver MNCs and splenocytes from *S. japonicum*-infected mice became IL-33-responsive and induced the production of IL-5 and IL-13. Since IL-33 exerts its cytokine activity through a signaling receptor known as ST2L, we examined ST2L expression in liver using mixed and single male infection model. It was determined that ST2L^+^ cells were upregulated on both splenocytes and liver MNCs during infection with mixed sex but not in mice with male-only infection, suggesting that higher ST2L was induced by SEA rather than by SWA.

IL-33 targets many immune cells involved in the pathogenesis of disease. Fort *et al*. described a population of non-T/non-B cells that released the type 2 cytokines IL-4, IL-5, and IL-13 in response to IL-25^24^. Later, several research groups^16^,^25^,^26^ reported the same populations (Lin^-^sca1^+^ Thy1^+^ST2L^+^) but named them differently (namely “natural helper cells”, “nuocytes”, and “innate helper type 2 cells”). Previous studies showed that IL-33 also targets T cells to amplify Th2 cytokines. Additionally, the macrophage is one of IL-33-responsive cells that can induce the production of IL-13. All these IL-33-responsive cells express ST2L and produce IL-5 and IL-13 in response to IL-33. We explored ST2L expression during *S. japonicum* infection and found that the dominant cells which responded to IL-33 were macrophages though many cells (including T cells, B cells and non-T/non-B cells) in the liver also expressed ST2L and responded to IL-33. Consistent with high ST2L expression on macrophages, the liver MNCs produced higher level of IL-13 when there are a higher number of macrophages. Of interest, are the differences in the response to IL-33 between mixed and male only-infection. IL-33 protein expression levels were enhanced both after mixed infection and male-only infection while the liver cells of the latter displayed a poor response to IL-33 and a reduction in Th2 cytokine production. Comparison of ST2L expression in LMNC reveals that ST2L^+^ macrophages are characteristic of the higher levels of IL-13 production during mixed infection. Thus, increased expression of ST2L expressed in the liver correlated with more severe type 2 inflammatory responses during *S. japonicum* infection.

Schistosome eggs induce AAM-rich granuloma and Th2-biased immunity that prevents acute mortality but ultimately causes liver fibrosis^5^. IFN-γ and TLR pathways orient macrophage function towards the M1 phenotype whereas IL-4 and IL-13 polarize macrophages function towards the M2 phenotype. Recently, several reports demonstrated that IL-33/ST2L also plays important roles in the amplification of M2 polarization^12^,^27^. Therefore, in the micro-environmental milieu is an important determinant of macrophage function during inflammation. We wondered whether SEA could polarize macrophage into AAM. Therefore, we cultured liver MNCs with IL-33 or SEA *in vitro* and found that both IL-33 and soluble egg antigen (SEA) increased the expression of ST2L on macrophages in contradistinction to SWA. Further analyses demonstrated that ST2L^+^ macrophages displayed the MHCII^-^CD11b^+^ M2 phenotype, indicating that IL-33 and SEA could enhance AAM polarization. Furthermore, more ST2L^+^ macrophages were observed in the liver and spleen at week 7 than at week 4 post infection and there were more ST2L^+^ macrophages in the liver of mixed infection than male-only infection at weeks 4, 7, and 10 post infection. Zhu *et al*. showed that SWA favors the generation of M1 macrophages, whereas SEA preferentially promotes M2-polarized phenotype during *S. japonicum* infection^28^. Taken together, SEA could upregulate ST2L expression and promotes macrophage responsive to IL-33 to produce IL-13. Both IL-13 and IL-33 further promote the polarization of macrophages toward M2 phenotype. Therefore, more and more ST2L^+^M2 macrophages appear in the liver. In addition, the deletion of macrophage in *S. japonicum* infected mice resulted in down-regulation of ST2L expression in T cells and decreased IL-13 production. These data indicate that IL-33 promotes type 2 immunity and helminth-induced hepatic granuloma pathology through induction of M2 macrophages. It should be noted that since SEA and IL-33 increase expression of ST2L it raises a question as to whether both have a synergistic or additive effect. Alternatively, SEA may induce IL-33 expression in hepatic cells and ST2L expression in macrophages. The delineation of these pathways remains to be seen.

In summary, we have demonstrated an important role for IL-33/ST2L-mediated type 2 immune responses and inflammation in the liver during *S. japonicum* infection. Selective ablation of IL-33 with a consequent diminution of inflammatory response in the liver may represent a potential therapeutic approach to hepatic granuloma pathology caused by *S. japonicum.*

## Methods

### Patients

Human studies were carried out in accordance with The Code of Ethics of the World Medical Association (Declaration of Helsinki) and approved by the Human Research Ethics Committee of the Sun Yat-sen University in China. The patients received an explanation regarding the scope of the study, such as objectives, procedures, and potential risks, and signed an informed consent statement before inclusion in the study. Sera were collected from patients with acute schistosomiasis who had contact with infected water, had eggs in the stool and a typical clinical manifestation.

### Parasite preparation and infection of mice

BALB/c mice (6 to 8 weeks old) were obtained from the Center of Experimental Animals, Sun Yat-sen University (Guangzhou, China). Animal protocols were approved by the Animal Care and Use Committee of Sun Yat-sen University. All animal experiments were performed in accordance with Chinese animal protection laws and with permission from the Institutional Review Board. *Oncomelania hupehensis* harboring *S. japonicum* cercariae (Chinese mainland strain) were purchased from the Jiangsu Institute of Parasitic Diseases (Wuxi, China). Mice were percutaneously infected with male cercariae for worm-only infections or male and female cercariae for egg-laying infections by placing a glass slide carrying 20 ± 2 *S. japonicum* cercariae on their abdomen for 15 minutes. After 4, 7, and 10 weeks, mice were sacrificed to obtain their livers and spleen (*n* = 4–6/group). SWA and SEA were prepared and diluted in PBS (pH 7.2) to a final concentration of 10 mg/mL, as previously described^25^,^26^. The protein concentration of SEA and SWA was determined using a bicinchoninic acid (BCA) Protein Assay kit (BioRad, Richmond, CA). Liver MNCs were isolated from the livers of mice using modifications of procedures previously described^28^. Briefly, mouse livers were minced and re-suspended in PBS (pH 7.2) and centrifuged at 50 × g for 5 minutes. Supernatants containing liver MNCs were collected and washed in PBS. The cell pellets were re-suspended in 37% Percoll solution (Sigma-Aldrich) and gently overlaid onto 70% Percoll. After centrifuged (850 × g) with the off-brake setting for 30 minutes at room temperature, the inter-phase containing liver MNCs were collected and washed twice in PBS. These cells were subsequently used for stimulation and surface staining.

### *In vitro* cell culture

Splenocytes and liver MNCs from infected mice were cultured in the presence or absence of SWA (50 μg/ml), SEA (20 μg/ml), IL-33 (10 ng/ml R&D Systems, Minneapolis, MN, USA) or Con A (5 μg/ml) in 96-well flat bottom plates. Cells were cultured at a density of 5 × 10^5^ cells/well in RPMI 1640 supplemented with 10% fetal calf serum, 2 mM L-glutamine, 100 U/ml penicillin, 100 ug/mL streptomycin, and 50 μM β-mercaptoethanol for 72 h. Supernatants were stored at −20 °C for cytokine analysis.

### Flow cytometry

Freshly isolated liver MNCs and spleen cells were initially incubated with anti-mouse CD16/32 (1:100 final dilution; Becton Dickinson) for 15 minutes at room temperature. The washed cells were incubated for 30 minutes at 4 °C with the following fluorescently labeled monoclonal antibodies: anti-ST2L-PE (eBioscience, San Diego, CA), anti-CD11b-APC (eBioscience), anti-CD19-PE-Cy7 (eBioscience), anti-CD4-PerCP (eBioscience), anti-CD3-APC (BD Pharmingen, San Diego, CA), anti-F4/80-FITC (eBioscience), anti-F4/80-PerCP-Cy5.5 (eBioscience), anti-MHCII-FITC (eBioscience). FACS analysis was performed using FACScalibur and/or FACSverse (both from Becton Dickinson).

### Quantitative RT-PCR

Total RNA was isolated from individual liver for analysis, using TRIzol (Invitrogen, Carlsbad, CA) according to the manufacturer’s recommendations and quantitated by using LightCycler480 real-time PCR System (Roche Diagnostics Inc). One microgram of total RNA was reverse transcribed to produce cDNA, and the cDNA was amplified using SYBR green Master Mix (Bio-Rad, Hercules, CA) as suggested by the manufacturer. The primer sequences used to amplify the mouse genes were as follows: ST2L forward, 5′-CAT GGC ATG ATA AGG CAC AC-3′; ST2L reverse, 5′-GTA GAG CTT GCC ATC GTT CC-3′; IL-13 forward, 5′-TCT TGC TTG CCT TGG TGG TC-3′; IL-13 reverse, 5′-GGT CTT GTG TGA TGT TGC TCA GC-3′; IL-5 forward, 5′-ATG GAG ATT CCC ATG AGC AC-3′; IL-5 reverse, 5′-CCC ACG GAC AGT TTG ATT CT-3′; IL-10 forward, 5′-TCG AAA TGA AAG TTC CAG CA–3; IL-10 reverse, 5′-CAT GGC ATG ATA AGG CAC AC-3′; CCL24 forward, 5′-GCT CTG CTA CGA TCG TTG-3′; CCL24 reverse 5′-AGC AAA CTT GGT TCT CAC TG-3′; Fizz1 forward, 5′-CTG CCC TGC TGG GAT GAC T-3′; Fizz1 reverse 5′-CAT CAT ATC AAA GCT GGG TTC TCC-3′; Ym1 forward 5′-CAA GTT GAA GGC TCA GTG GCT C-3′; YM1 reverse 5′-CAA ATC ATT GTG TAA AGC TCC TCT C-3′; β-actin forward: 5′-GGC ATC CTG ACC CTG AAG TA-3′; reverse: 5′-CTC TCA GCT GTG GTG GTG AA-3′. Relative mRNA levels were calculated after normalization to the housekeeping gene β-actin endogenous control and fold expression was measured by comparison to the expression of the uninfected controls.

### ELISA assay

Enzyme-linked immunosorbent assay (ELISA) was performed to measure the production of cytokines in cell culture supernatants and in the sera of mice. IL-5, IL-13, and IL-33 were measured by using ELISA kits (eBioscience, San Diego, CA) according to the manufacturer’s instructions.

### Histopathology

Formalin-fixed, paraffin-embedded liver tissues from uninfected controls and mice infected at 4, 7, and 10 weeks post infection were sectioned and stained with H&E to assess histopathology including granuloma formation.

### Macrophage depletion

Macrophage depletion studies were done in *Balb/c* mice using liposome-encapsulated clodronate injection as previously described^21^,^27^. Mice were percutaneously infected with male and female cercariae by placing a glass slide carrying 20 ± 2 *S. japonicum* cercariae on their abdomen for 15 minutes. From weeks 4 to 6, mice received 4 tail vein injections of 100 μL/10g body weight of 1 mg/ml PBS or clodronate (CLOD)-containing liposomes. Mice were sacrificed 24 hours after the fourth injection, at which time plasma, spleen and liver tissue were collected.

### Statistical analysis

All statistics were carried out using Prism (GraphPad Software), and *P* values were obtained by using two-tailed Student’s t-test for two group comparisons. The data were expressed as mean ± SEM or mean ± SD. Values of p < 0.05 were considered as statistically significant.

## Additional Information

**How to cite this article**: Peng, H. *et al*. IL-33 Contributes to *Schistosoma japonicum*-induced Hepatic Pathology through Induction of M2 Macrophages. *Sci. Rep.* **6**, 29844; doi: 10.1038/srep29844 (2016).
