# Erratum: IL-33 Contributes to *Schistosoma japonicum*-induced Hepatic Pathology through Induction of M2 Macrophages

**DOI:** 10.1038/srep39568

**Published:** 2017-01-04

**Authors:** Hui Peng, Qixian Zhang, Xiaojuan Li, Zhen Liu, Jia Shen, Rui Sun, Jie Wei, Jia Zhao, Xiaoying Wu, Feng Feng, Shuping Zhong, Xi Sun, Zhongdao Wu

Scientific Reports 6: Article number: 2984410.1038/srep29844; published online: 07 21 2016; updated: 01 04 2017

In this Article, there are errors in the affiliations for Qixian Zhang and XiaoJuan Li. The correct affiliations are listed below

Qixian Zhang

Key Laboratory of Tropical Disease Control (Sun Yat-sen University), the Ministry of Education PRC, Guangzhou, 510080, China.

Zhongshan School of Medicine, Sun Yat-sen University, 74 Zhongshan 2nd Road, Guangzhou, 510080, China.

The Third Affiliated Hospital of Sun Yet-sen University, Guangzhou, 510630, China.

XiaoJuan Li

School of Pharmaceutical Sciences, Southern Medical University, 1838 North Guangzhou Avenue, Guangzhou, 510515, China.

